# Chilling Complications: A Case of Cryoglobulinemic Vasculitis in a Patient With Newly Diagnosed Sjögren’s Syndrome

**DOI:** 10.7759/cureus.105707

**Published:** 2026-03-23

**Authors:** Alexandra Anderson, Arya Loghmani, Dani'elle Despanie, Barrett Ford

**Affiliations:** 1 Rheumatology, Louisiana State University Health Sciences Center, New Orleans, USA

**Keywords:** autoimmune vasculitis, cryoglobulinemia, cryoglobulinemia vasculitis, rituximab use in sjogren, sjogrens syndrome

## Abstract

In rare circumstances, Sjögren’s syndrome can cause systemic illness in the form of cryoglobulinemic vasculitis (CV). Diagnostic workup is of the utmost importance to prevent severe end-organ damage and even death, especially in the case of rapidly progressive disease.

In this case, a 47-year-old male presented to the hospital with shortness of breath, unilateral leg swelling, and a lower extremity wound worsening over a three-month period. During this admission, the patient was found to have diffuse lymphadenopathy with a negative workup for malignancy. The patient met the criteria for Sjögren’s syndrome and was initiated on outpatient disease-modifying antirheumatic drug (DMARD) therapy. The patient was readmitted to the hospital in undifferentiated shock and was found to have CV, with significant end-organ damage. He was treated with pulse-dose steroids, plasmapheresis, and rituximab infusions. After a lengthy three-month admission, he was finally discharged in stable condition. CV can present very similarly to antiphospholipid syndrome and antineutrophil cytoplasmic antibody (ANCA) vasculitis. Biopsies prove invaluable in the diagnosis. This manifestation of Sjögren’s syndrome is uncommon, and it is often difficult to assess which patients may be more at risk for the complication. This case illustrates the importance of early identification of patients with Sjögren’s syndrome who may be at risk for the development of vasculitis.

## Introduction

Sjögren’s syndrome is an autoimmune disease that typically affects exocrine glands, like the salivary glands, but can include multi-organ system involvement in some cases. Cryoglobulinemic vasculitis (CV) is a rare condition characterized by small vessel vasculitis, often associated with underlying infections such as hepatitis C or autoimmune diseases, including Sjögren’s syndrome. Cryoglobulins are insoluble immunoglobulins that precipitate at cold temperatures in the blood, leading to vascular damage and subsequent multi-organ involvement [1]. Manifestations of CV include arthralgias, purpura, and arthritis. Cryoglobulins are classified into groups based on the type of immunoglobulin, or antibody, whether monoclonal or polyclonal. Sjögren’s syndrome is usually associated with type 2 cryoglobulins, which are monoclonal immunoglobulins, typically either IgG or IgM, with rheumatoid factor [2]. In 1986, Tzioufas et al. [1] reported that one-third of individuals with primary Sjögren’s syndrome (pSS) have cryoglobulins based on the evaluation of a mixed group of monoclonal and polyclonal immunoglobulins in Sjögren’s patients using gel electrophoresis. This foundational research not only established the significant prevalence of cryoglobulin production in patients with pSS but also contributed to the emerging understanding of the pathogenic role these immune complexes play in vascular injury and the evolution of systemic vasculitic manifestations. Therefore, it can be difficult to diagnose this disease in a timely manner, when symptoms can progress very quickly from the time of initial Sjögren’s symptoms to systemic vasculitis manifestations [1].

The development of clinical vasculitis in individuals with Sjögren’s syndrome is rare. The prevalence of CV in pSS ranges from 3 to 10%. In a large multicenter retrospective study of individuals with pSS who developed CV, less than 10% of those with pSS developed CV. Of that group, more than half developed at least one feature of CV within the first year after their pSS diagnosis. Studying the characteristics and treatment responses of this subset of patients presents challenges due to its relatively low incidence. Various classifications of cryoglobulins are well established, but identifying them in patients with newly diagnosed Sjögren’s syndrome remains an area of ongoing research. CV is associated with potential end-organ damage and an elevated risk of developing lymphoma, which significantly increases morbidity and mortality [2]. CV is an uncommon manifestation of Sjögren’s syndrome that can be diagnostically challenging. Given its serious complications, early recognition and tailored therapeutic strategies are essential to improve patient outcomes and reduce long-term risks.

## Case presentation

A 47-year-old male with a past medical history of recurrent nephrolithiasis, coronary artery disease (CAD), left lower extremity (LLE) deep vein thrombosis (DVT), hypothyroidism, and severe chronic obstructive pulmonary disease (COPD) developed new-onset Raynaud’s phenomenon, sicca symptoms, and recurrent urticaria. These symptoms persisted for approximately 18 months before he experienced a three-month period of progressive LLE swelling, shortness of breath, and the development of a right lower extremity (RLE) ulcer, ultimately requiring hospitalization. 

**Figure 1:**
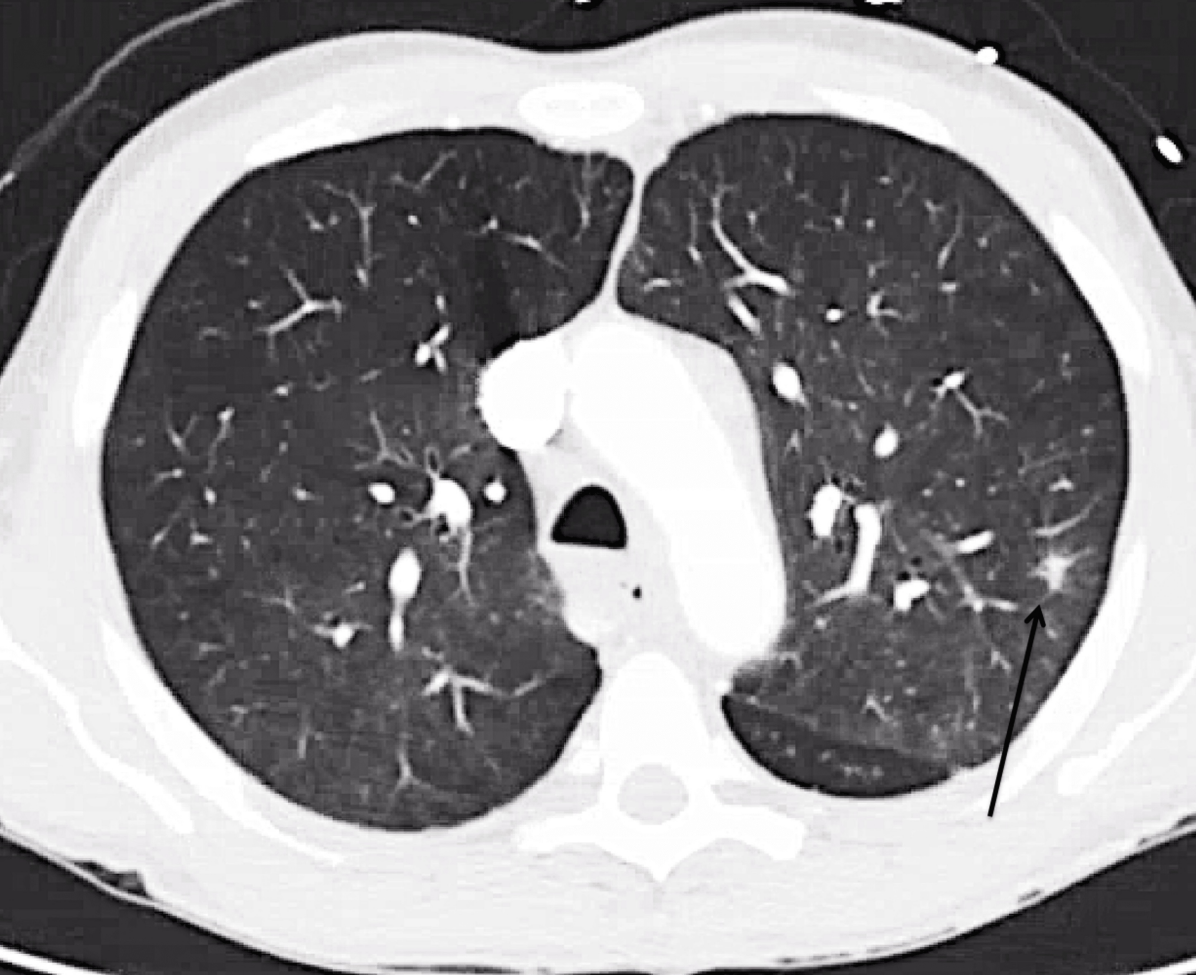
Computed tomography scan of the chest showing a spiculated lung nodule

**Figure 2:**
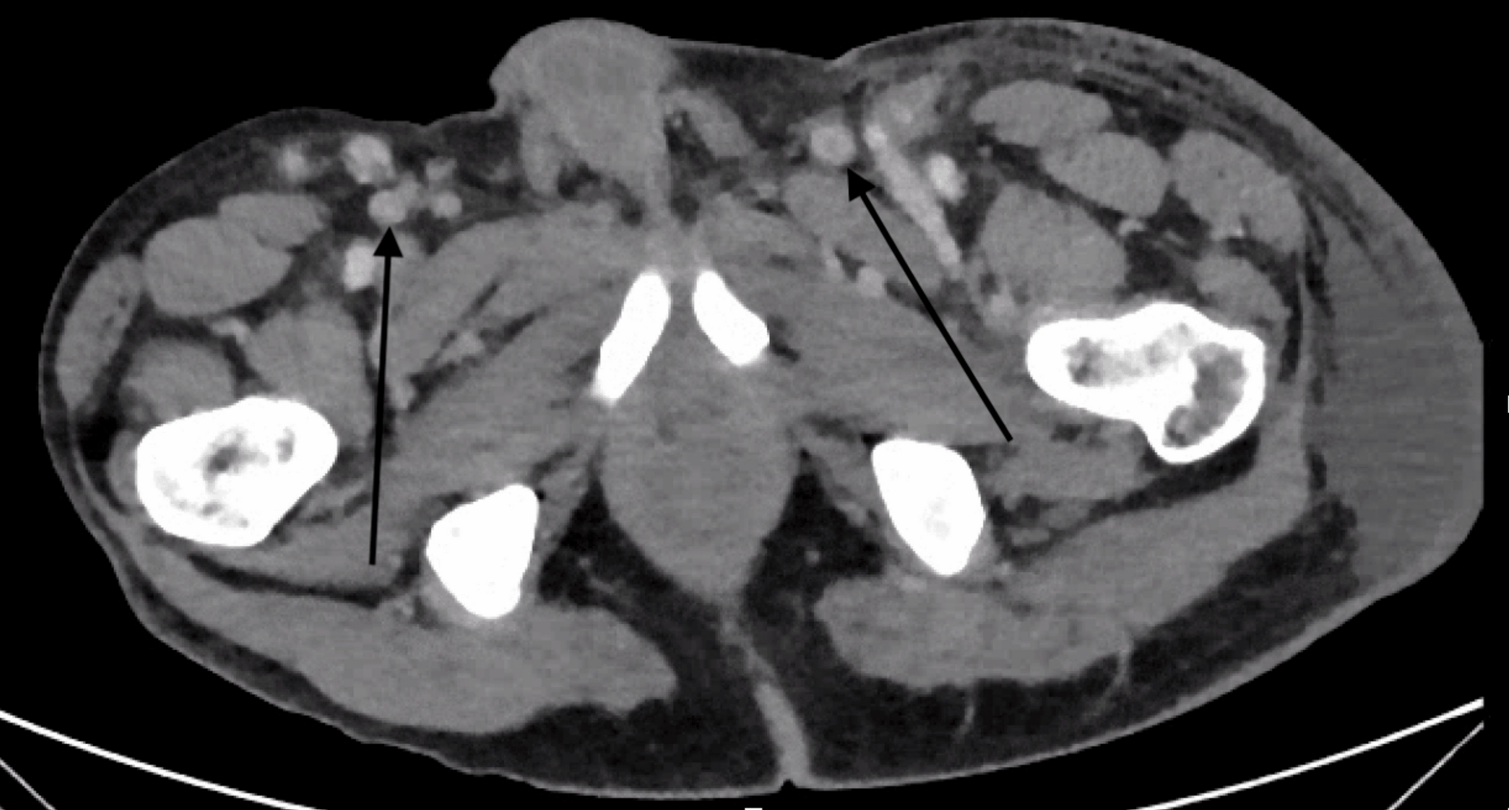
Computed tomography scan of the abdomen and pelvis showing inguinal lymphadenopathy

Computed tomography (CT) imaging revealed a 9 mm irregular spiculated opacity in the left upper lung lobe along with diffuse lymphadenopathy (LAD) involving the axillary, mediastinal, hilar, inguinal, pelvic sidewall, and periaortic regions (Figures 1-2). Laboratory studies revealed an erythrocyte sedimentation rate (ESR) of 17 mm/hour (reference range 0-15 mm/hour), C-reactive protein (CRP) of 5.8 mg/L (reference range: 0-3 mg/L), positive direct antinuclear antibody (ANA), SSA/SSB >8 units (reference range <7 units), and low complement levels, with C3 at 57 mg/dL (reference range: 83-180 mg/dL) and C4 at <8 mg/dL (reference range: 18-55 mg/dL). Additionally, there was detection of 3 mg/dL IgG and 2 mg/dL IgM cryoprecipitates (Table 1). The remainder of the autoimmune, infectious, and malignancy workup was unremarkable. A right axillary lymph node (LN) biopsy showed reactive follicular hyperplasia with dermatopathic lymphadenitis and no evidence of malignancy. The lung mass was not thought to be malignant when reviewed by the oncology team, and therefore, the lung mass was not biopsied. The patient was discharged with plans for close outpatient rheumatology follow-up.

**Table 1:** Initial miscellaneous laboratory values C3, complement 3; C4, complement 4; CRP, C-reactive protein; ESR, erythrocyte sedimentation rate; IgG cryoprecipitate, immunoglobulin G cryoprecipitate; IgM cryoprecipitate, immunoglobulin M cryoprecipitate; SSA, Ro antibody; SSB, La antibody

Test	Reference Range	Result
ESR	0-15 mm/hour	17 mm/hour
CRP	0-3 mg/L	5.8 mg/L
SSA/SSB	<7 units	>8 units
C3	83-180 mg/dL	57 mg/dL
C4	18-55 mg/dL	<8 mg/dL
IgG cryoprecipitate	0 mg/dL	3 mg/dL
IgM cryoprecipitate	0 mg/dL	2 mg/dL

At the initial rheumatology clinic visit, the physical exam was notable for poor dentition with decreased salivary pooling, bilateral axillary LAD, and a RLE wound with granulation tissue and surrounding erythema. Given the lung lesion and generalized LAD, malignancy was initially a primary concern. However, LN biopsy and further diagnostic evaluation were inconsistent with a neoplastic process. 

Despite a course of empiric antibiotics, the RLE wound progressed. Given the constellation of sicca symptoms, positive SSA/SSB antibodies, hypocomplementemia, and presence of cryoglobulins, there was a high clinical suspicion for Sjögren’s syndrome complicated by mixed CV. The patient was initiated on hydroxychloroquine and a slow prednisone taper. He was subsequently evaluated by dermatology, who performed three punch biopsies from different sites. Histopathology of the right upper extremity revealed perivascular neutrophilic collagenosis with early leukocytoclasia, consistent with evolving leukocytoclastic vasculitis (LCV). A right chest wall biopsy demonstrated LCV with occasional eosinophils. The right thigh biopsy showed mild lymphocytic vasculitis with focal small-vessel thrombosis vasculopathy. Unfortunately, the patient was lost to follow-up with rheumatology, limiting further diagnostic evaluation and longitudinal management. 

Several months later, the patient underwent an interventional radiology-guided LN biopsy, which demonstrated reactive lymphoid hyperplasia. Concurrent flow cytometry revealed no immunophenotypic evidence of a lymphoproliferative disorder. Rheumatology subsequently resumed treatment with hydroxychloroquine. 

**Figure 3:**
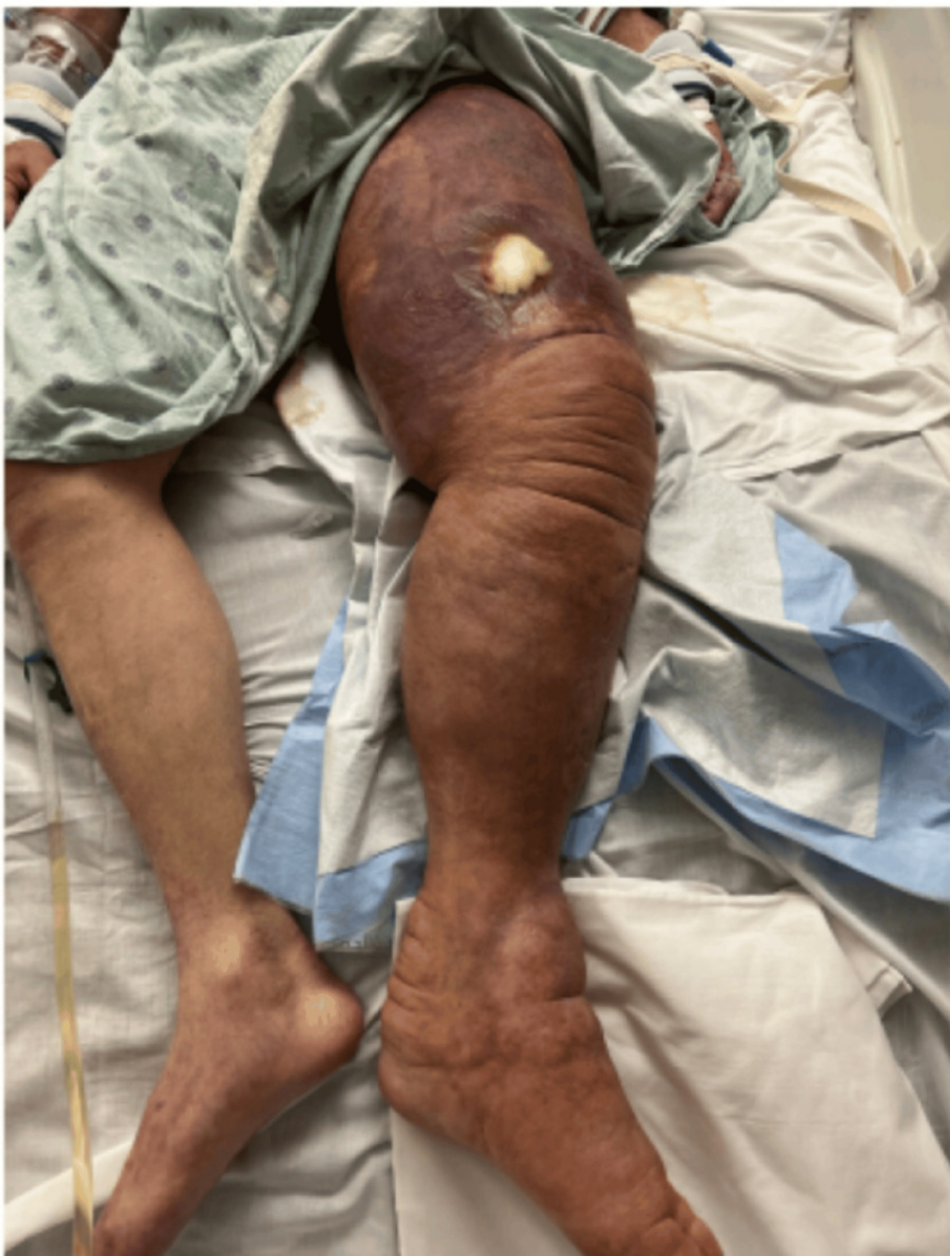
Left lower extremity edema and purpuric skin changes on the first day of admission

Several weeks later, the patient presented to the hospital with worsening dyspnea on exertion, progression of RLE ulcer, new-onset LLE pain and swelling, nausea, vomiting, diarrhea, and subjective fevers. CT imaging revealed a stable, diffuse LAD, a rounded spleen, and a borderline enlarged pulmonary artery. The previously noted 9 mm spiculated left upper lobe pulmonary opacity remained unchanged. He was admitted to the medical intensive care unit for multiorgan failure in the setting of undifferentiated shock and required endotracheal intubation for respiratory support. Rheumatology was consulted for further evaluation. Laboratory evaluation showed complement C3 at 32 mg/dL (reference range: 83-180 mg/dL), C4 at <8 mg/dL (reference range: 18-55 mg/dL), negative rheumatoid factor (RF), elevated soluble interleukin-2 receptor alpha at 5205 U/mL (reference range: 223-710 U/mL), and ferritin at 614.8 ng/mL (reference range: 20-300 ng/mL), as shown in Table 2. Given concern for catastrophic antiphospholipid syndrome (CAPS), therapeutic anticoagulation was initiated. On physical exam, his lower extremity wounds had worsened, with increasing erythema, edema, and purulence. In addition, worsening purpuric lesions and edema of LLE were noted, as seen in Figure 3. Blood cultures returned positive for *Streptococcus pneumoniae*. Due to high clinical suspicion for necrotizing fasciitis of the LLE, he was taken emergently to the operating room for incision and drainage. Pathology revealed acute vasculitis of medium to large caliber blood vessels, dermal edema with acute abscess and chronic inflammation, necroinflammatory debris, and fibroconnective tissue degeneration. Intraoperative findings revealed healthy, viable muscle with intact fascia. Tissue cultures from the site also grew *Streptococcus pneumoniae*.

**Table 2:** Miscellaneous lab values on later admission C3, complement 3; C4, complement 4; IL-2 RA, interleukin-2 receptor alpha

Test	Reference Range	Result
C3	83-180 mg/dL	32 mg/dL
C4	18-55 mg/dL	<8 mg/dL
IL-2 RA	223-710 U/mL	5205 U/mL
Ferritin	20-300 ng/mL	614.8 ng/mL

During his hospitalization, he developed a non-ST elevation myocardial infarction (NSTEMI) with peak troponin levels exceeding 50 ng/mL. Transthoracic echocardiography (TTE) demonstrated global hypokinesis with a reduced ejection fraction of 25% as well as right ventricular mid-wall hypokinesis with preserved apical function (Video 1). Pulmonary thromboembolism was excluded with a CT pulmonary embolism scan. Left and right heart catheterization revealed cardiogenic shock with biventricular failure. Coronary angiography showed 40-50% stenosis of the left main coronary artery stenosis but no evidence of obstructive CAD (Video 2). 

**Video 1:** Transthoracic echocardiogram showing global hypokinesis

**Video 2:** Left heart catheterization

Considering these findings and ongoing hemodynamic instability, there was a high clinical suspicion of myocarditis. He was initiated on intravenous methylprednisolone 40 mg twice daily. Given concern for concurrent infection, an initially conservative steroid regimen was chosen. However, due to progressive clinical decline, methylprednisolone was later escalated to 250 mg twice daily for three days. He also developed acute kidney injury with progressive renal failure, requiring initiation of continuous renal replacement therapy (CRRT). During this time, his white blood cell count rose to more than 60,000/μL, potentially secondary to a leukemoid reaction. However, testing for BCL-ACL and JAK2 returned negative. 

**Figure 4:**
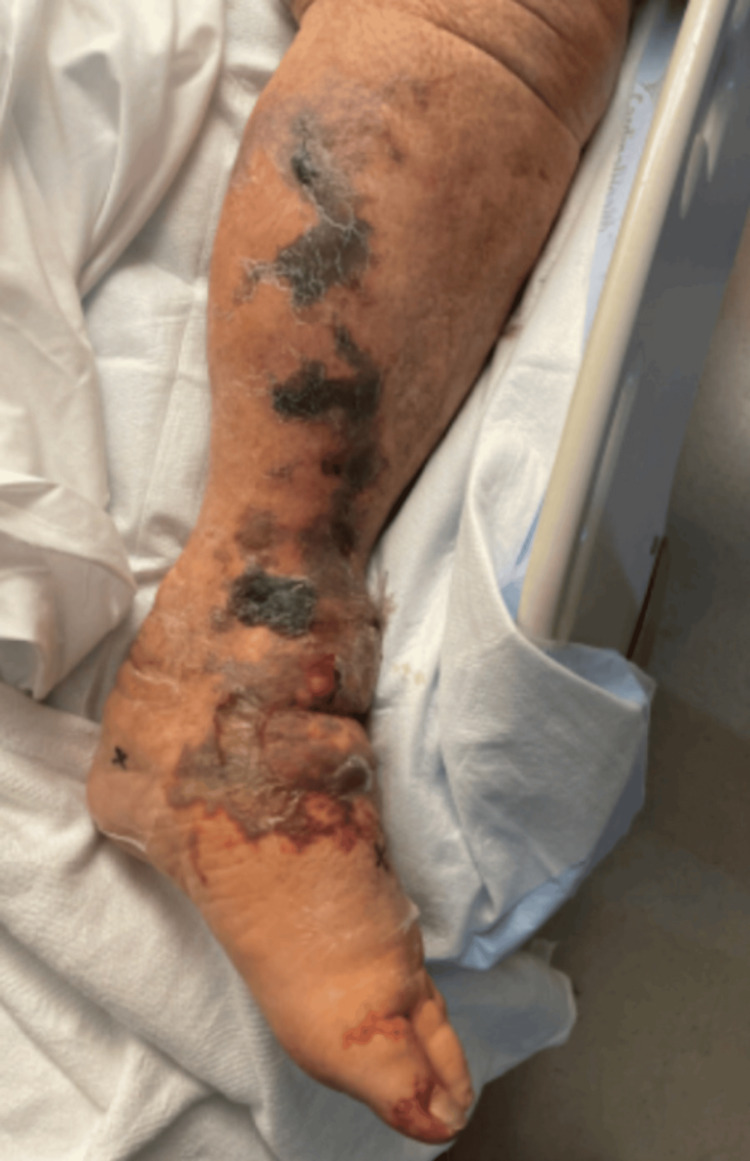
Left lower extremity necrotic lesions

**Figure 5:**
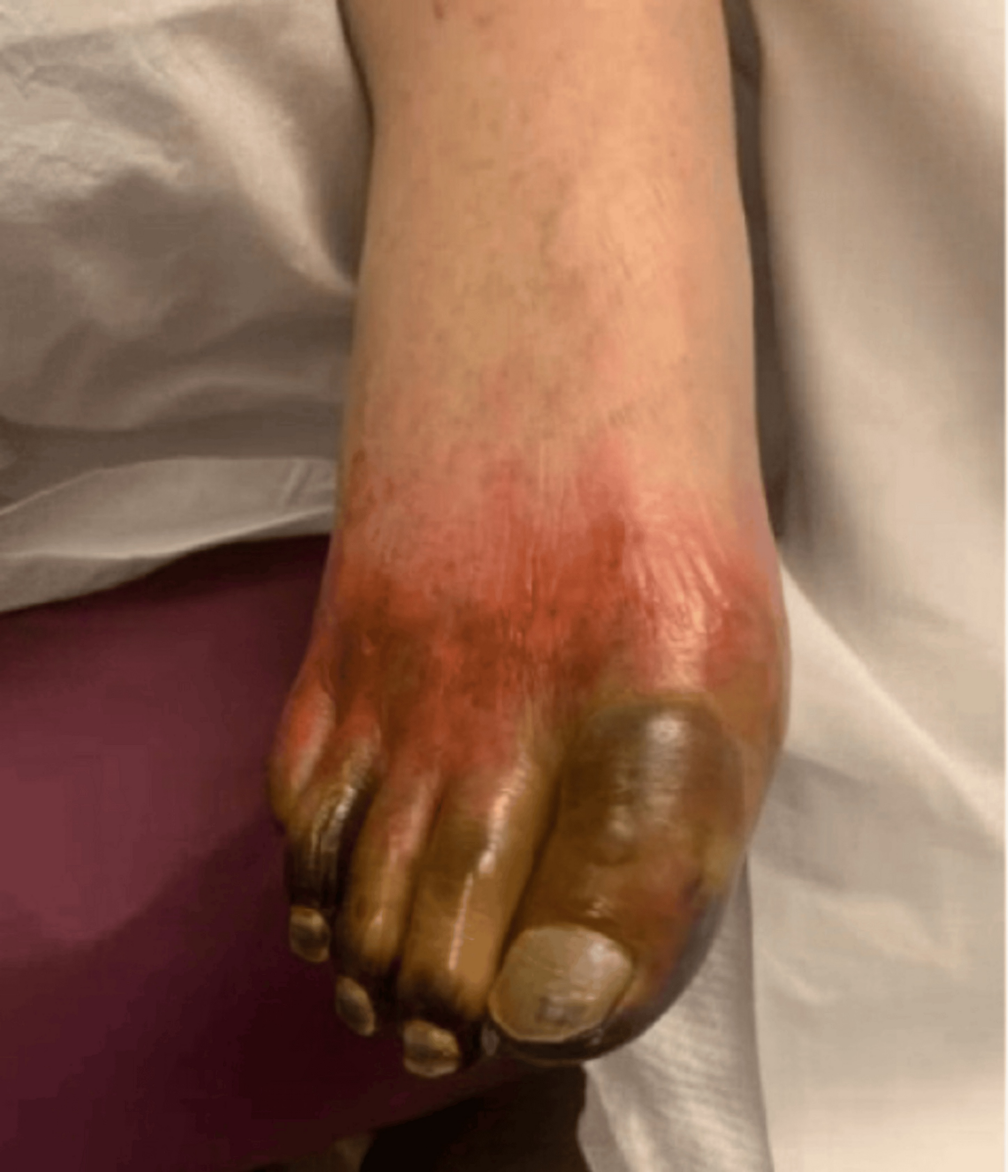
Right foot with necrosis of toes

The patient was successfully weaned off vasopressor support and started producing urine. However, his clinical course was complicated by the development of worsening purpuric lesions and necrosis as seen in Figures 4-5. Further rheumatologic evaluation revealed a positive antineutrophil cytoplasmic antibody (ANCA) titer of 1:160 with an inconclusive pattern, elevated anti-MPO antibodies at 38 AU/mL (reference range 0-19 AU/mL), and negative antiphospholipid antibodies (aPL). A skin biopsy from the left forearm demonstrated LCV, with direct immunofluorescence (DIF) showing vascular deposition of IgA, IgG, IgM, C3, and fibrinogen.

Plasma exchange (PLEX) therapy was initiated, with a total of five treatments administered. Despite this, skin lesions on the LLE continued to worsen, accompanied by new-onset fevers. He was taken to the operating room for incision and drainage of the LLE. Pathology revealed acute vasculitis involving medium to large caliber vessels, dermal edema with mixed acute and chronic inflammation, necroinflammatory debris, and degeneration of fibroconnective tissue. 

Hospital course was further complicated by *Candida albicans* fungemia, with transesophageal echocardiography (TEE) identifying a 1.6 × 1.6 cm mass invading the anterolateral papillary muscle with multiple, smaller hyperechoic lesions throughout the interventricular septum. A six-week course of antifungal therapy was initiated. Thiopurine methyltransferase (TPMT) testing was normal, allowing for initiation of azathioprine in addition to ongoing intravenous methylprednisolone. Despite repeat incision and drainage of the LLE, the patient ultimately required a left below-the-knee amputation and a right transmetatarsal amputation due to progressive tissue necrosis. He was unable to be weaned from mechanical ventilation secondary to critical illness myopathy and subsequently underwent tracheostomy placement. 

The patient developed recurrent undifferentiated shock, prompting TTE, which revealed a large pericardial effusion with pre-tamponade physiology. An emergent pericardiocentesis was performed. Shortly thereafter, he developed progressive duskiness of the bilateral upper extremities, and PLEX was resumed at a frequency of two treatments per week. He again developed undifferentiated shock, and a TTE was performed, revealing a large pericardial effusion with pre-tamponade physiology, so an emergent pericardiocentesis was performed. He started to develop duskiness over the bilateral upper extremities, so he was placed back on PLEX for two treatments a week. He subsequently required multiple surgical interventions, including incision and drainage, revision of prior amputations, and autografting of the left thigh. A multidisciplinary meeting was arranged, and the burn team recommended deferring B-cell depletion therapy until at least two weeks after the completion of the final amputation and revision procedures. 

The hospital course was further complicated by *Klebsiella pneumoniae* bacteremia associated with shock. A CT scan of the abdomen and pelvis was obtained to evaluate for the source, which demonstrated global splenic infarction with subsequent splenic parenchymal liquefaction. A CT scan of the femur revealed a left tibial fluid collection and evidence of DVT involving multiple deep venous structures.

Due to persistent hypotension, the endocrinology team was consulted and initiated stress dose hydrocortisone and fludrocortisone for suspected adrenal insufficiency. After completion of antibiotic therapy, intravenous rituximab was initiated at a dose of 500 mg every two weeks for a total of two doses. Corticosteroids were subsequently transitioned from intravenous to oral prednisone. Unfortunately, the patient was found to be colonized with *Candida auris*. Eventually, he was discharged to an inpatient rehabilitation facility. 

The patient was continued on rituximab therapy for several months; however, treatment was interrupted due to difficulties with insurance authorization. As a result, he was transitioned to oral cyclophosphamide at a dose of 150 mg daily. Unfortunately, he was lost to follow-up for over a year and did not undergo routine laboratory monitoring during this time. 

He later presented with symptoms of poor appetite, chills, night sweats, hemoptysis, and unintentional weight loss, despite adherence to cyclophosphamide. Laboratory evaluation revealed a hemoglobin of 5.1 g/dL and a platelet count of 26 × 10⁹/L. He was directed to the emergency department and admitted for evaluation of pancytopenia. Following an extensive workup, the cytopenias were attributed to oral cyclophosphamide toxicity, and the medication was discontinued. Rituximab therapy was re-initiated and has been continued for over a year. The dosing was recently adjusted to 1 g every six months. The patient has otherwise remained clinically stable and is doing well.

## Discussion

CV is a rare multisystem disease characterized by cutaneous and sometimes multiorgan manifestations. Individuals with CV typically develop purpura, generalized weakness, and arthralgias. The workup generally includes screening for hepatitis infections, autoimmune disorders, and malignancy. The classification criteria for CV are largely based upon the detection of serum cryoglobulins in addition to low serum complement and clinical symptoms. As of 2011, a large, multicenter European study, commonly referred to as the De Vita criteria, proposed a standardized set of classification criteria for the diagnosis of CV. This initiative, supported by the European Vasculitis Study Group (EUVAS) and the Italian Group for the Study of Cryoglobulinemia, aimed to improve diagnostic consistency across clinical settings. According to the criteria, patients were required to meet at least two of the clinical history or questionnaire-based criteria, which included a history of viral hepatitis (specifically hepatitis C) and the presence of petechiae or palpable purpura. In addition, patients needed to exhibit at least three clinical manifestations such as fatigue, fever, arthralgias, arthritis, purpura, ulcers, necrotizing vasculitis, Raynaud’s phenomenon, peripheral neuropathy, cranial nerve involvement, or central nervous system (CNS) vasculitis. Diagnostic confirmation also required fulfillment of at least two laboratory criteria, which included hypocomplementemia (low serum C4), a positive rheumatoid factor, or the presence of a serum monoclonal component (M-protein) [3]. 

There are several possible underlying causes for CV. The patient was initially screened for infectious causes, which yielded negative results for hepatitis, tuberculosis, and HIV. Malignancy workup was significant for diffuse LAD with suspicion for lymphoma, as well as a spiculated lung nodule representing a possible neoplasm. An excisional axillary LN biopsy was obtained inpatient, which revealed reactive follicular hyperplasia and was negative for malignancy. Rheumatologic workup was significant for positive ANA, elevated anti-SSA and anti-SSB, and low C3 and C4 levels. He was ultimately diagnosed with Sjögren’s syndrome based on his positive SSA antibodies, chronic dry eye, chronic dry mouth, and ocular staining score of greater than 5. These features meet the scoring criteria for a total of four items outlined in the 2016 American College of Rheumatology (ACR)-European League Against Rheumatism (EULAR) Classification Criteria for Primary Sjögren’s Syndrome [4].

At the onset of hospitalization, the primary diagnostic concern was antiphospholipid syndrome (APS), a prothrombotic autoimmune condition characterized by the presence of aPL that promotes endothelial dysfunction and intravascular coagulation, leading to arterial and/or venous thrombosis. CAPS - a rare but severe variant - was considered. CAPS is marked by rapidly progressive, widespread thrombotic microangiopathy resulting in multiorgan failure, most often manifesting with occlusions in large vessels, though it may mimic other systemic vasculitides. Comprehensive aPL testing, including lupus anticoagulant, anti-cardiolipin antibodies, and anti-β2 glycoprotein I antibodies, was negative. Since the presence of at least one positive aPL test is a required component of the classification criteria for APS and CAPS, this seronegative status strongly argues against the diagnosis. Skin biopsy from the left forearm demonstrated LCV with positive DIF for IgA, IgG, IgM, C3, and fibrinogen within vessel walls. This pattern is indicative of immune complex-mediated vasculitis, such as CV, rather than the pauci-immune thrombotic microangiopathy characteristic of CAPS. No evidence of fibrin thrombi or endothelial cell injury due to thrombosis was observed. CAPS typically presents with acute, simultaneous involvement of three or more organ systems within a period of days [5]. In this case, the disease progression was subacute and primarily involved the skin and peripheral vasculature, without the fulminant, systemic organ dysfunction required for a CAPS diagnosis. 

In parallel, ANCA-associated vasculitis (AAV) was also considered, given the presence of a positive ANCA tier (1:160) and elevated anti-myeloperoxidase (MPO) antibodies (38 AU/mL) [6]. However, this serologic finding was deemed non-diagnostic for several reasons. The ANCA pattern was indeterminate, and the MPO antibody level, while mildly elevated, was not in the range typically seen with active AAV. Low-level MPO positivity may occur in various autoimmune or inflammatory conditions and is not, in isolation, sufficient to diagnose AAV. AAV is defined by pauci-immune necrotizing vasculitis, which shows minimal or absent immune complex deposition on immunofluorescence. In contrast, the patient’s biopsy showed robust immune complex deposition, a hallmark of CV. While CV most commonly involves small to medium-sized vessels, rare cases have been documented involving larger vessels, which may explain the atypical histological finding of medium to large vessel vasculitis in this patient. AAV typically involves small to medium vessels and is often accompanied by necrotizing glomerulonephritis or pulmonary capillaritis - features not observed in this case [7]. 

In summary, both CAPS and AAV were considered early in the diagnostic workup. However, CAPS was ruled out based on negative antiphospholipid serologies, absence of histologic thrombosis, and lack of rapidly progressive multiorgan failure. Similarly, AAV was considered unlikely due to non-specific ANCA-MPO results, the presence of immune complex deposition on biopsy, and an overall clinical picture more consistent with CV, albeit with some atypical features such as involvement of larger-caliber vessels. These findings collectively support the diagnosis of CV as the most unifying explanation for this patient’s presentation. 

Due to the various underlying disorders as well as the clinical manifestations, pharmacological therapy can be quite challenging. The first-line treatment for moderate to severe CV involves high-dose steroids and rituximab. A multicenter, randomized controlled trial showed that Rituximab therapy was superior to standard treatments such as corticosteroids, cyclophosphamide, or plasmapheresis in those with HCV-related CV [8]. A longitudinal study of 13 patients with pSS and CV who were followed from 1999 to 2023 also demonstrated that rituximab was very effective in controlling vasculitis manifestations [9]. In cases of critical illness, pulse dose steroids and plasmapheresis are usually started first. Usually, this is done preceding rituximab or cyclophosphamide. These immunosuppressants may be continued long-term to prevent relapse of symptoms [10]. For those with hepatitis infection, anti-viral treatments can be combined with agents like rituximab [8]. A 2008 randomized study of 17 patients with HCV-related CV were assigned to receive immunosuppressive therapy with or without apheresis over a 12-week period. Immunosuppressive therapy included options such as interferon therapy, prednisone, cyclophosphamide, ribavirin, and cyclosporin. Those who received immunosuppressive therapy along with apheresis had better overall clinical scores, indicating better signs and symptoms, compared to those who only received immunosuppressive therapy [11]. Overall, the combination of steroids, plasmapheresis, and immunosuppressant therapy has yielded better outcomes overall for patients with CV rather than just plasmapheresis on its own. 

Interventions for the patient described above included initiation of IV steroids prior to transitioning to oral steroids. Plasmapheresis was also performed several times during hospitalization. Prior to discharge, the patient was initiated on rituximab. After discharge, the patient’s rituximab coverage was denied by insurance, and he had to switch to oral cyclophosphamide. He subsequently developed generalized B symptoms, and cyclophosphamide was discontinued due to the development of treatment-related cytopenias, which were not promptly identified or managed as the patient was lost to follow-up. After discontinuing cyclophosphamide treatment, the patient was reinitiated on rituximab. He is currently well controlled on rituximab as a maintenance therapy. 

## Conclusions

This report highlights a clinically significant case of CV emerging soon after the diagnosis of Sjögren’s syndrome, underscoring a potential link between the two conditions that warrants further investigation. The relatively rapid onset of vasculitis in this patient raises important questions about the pathophysiological mechanisms involved and the possible existence of predisposing factors that may accelerate or trigger such complications in a subset of individuals with Sjögren’s syndrome.

Given the potential severity and systemic nature of CV, identifying those at heightened risk is crucial for early intervention and improved outcomes. Future research should aim to uncover biomarkers, genetic predispositions, or environmental triggers that contribute to this association. A better understanding of these risk factors would not only enhance early detection but also facilitate the development of targeted treatment strategies. Ultimately, this case emphasizes the importance of vigilance in patients newly diagnosed with Sjögren’s syndrome and supports the need for a multidisciplinary approach to management, combining rheumatologic and immunologic expertise to improve patient care and long-term prognosis.
